# Oral Supplementation of Sodium Butyrate Attenuates the Progression of Non-Alcoholic Steatohepatitis

**DOI:** 10.3390/nu12040951

**Published:** 2020-03-30

**Authors:** Anja Baumann, Cheng Jun Jin, Annette Brandt, Cathrin Sellmann, Anika Nier, Markus Burkard, Sascha Venturelli, Ina Bergheim

**Affiliations:** 1 Department of Nutritional Sciences, Molecular Nutritional Science, University of Vienna, Althanstraße 14, UZA II, 1090 Vienna, Austria; anja.baumann@univie.ac.at (A.B.); annette.brandt@univie.ac.at (A.B.); anika.nier@univie.ac.at (A.N.); 2 Institute of Nutritional Sciences, SD Model Systems of Molecular Nutrition, Friedrich-Schiller-University Jena, Dornburger Straße 22-25, 07743 Jena, Germany; taiji-2002@hotmail.com (C.J.J.); CathrinSellmann@gmx.de (C.S.); 3 Institute of Physiology, Department of Vegetative and Clinical Physiology, University Hospital Tuebingen, Wilhelmstraße 56, 72074 Tuebingen, Germanysascha.venturelli@uni-hohenheim.de (S.V.); 4 Institute of Biological Chemistry and Nutrition, University of Hohenheim, Garbenstraße 30, 70599 Stuttgart, Germany

**Keywords:** inducible nitric oxide synthase, melatonin synthesis, non-alcoholic steatohepatitis, sodium butyrate, toll-like receptor 4

## Abstract

Sodium butyrate (SoB) supplementation has been suggested to attenuate the development of non-alcoholic fatty liver disease (NAFLD). Here, we determined the therapeutic potential of SoB on NAFLD progression and molecular mechanism involved. Eight-week old C57BL/6J mice were pair-fed a fat-, fructose- and cholesterol-rich diet (FFC) or control diet (C). After 8 weeks, some mice received 0.6g SoB/kg bw in their respective diets (C+SoB; FFC+SoB) or were maintained on C or FFC for the next 5 weeks of feeding. Liver damage, markers of glucose metabolism, inflammation, intestinal barrier function and melatonin metabolism were determined. FFC-fed mice progressed from simple steatosis to early non-alcoholic steatohepatitis, along with significantly higher TNFα and IL-6 protein levels in the liver and impaired glucose tolerance. In FFC+SoB-fed mice, disease was limited to steatosis associated with protection against the induction of *Tlr4* mRNA and iNOS protein levels in livers. SoB supplementation had no effect on FFC-induced loss of tight junction proteins in the small intestine but was associated with protection against alterations in melatonin synthesis and receptor expression in the small intestine and livers of FFC-fed animals. Our results suggest that the oral supplementation of SoB may attenuate the progression of simple steatosis to steatohepatitis.

## 1. Introduction

Studies suggest that the global prevalence of non-alcoholic fatty liver disease (NAFLD) in the general population is ~25% [1]. NAFLD encompasses a large spectrum of diseases, including simple hepatic steatosis, steatohepatitis (NASH), hepatic fibrosis, and cirrhosis and even hepatocellular carcinoma [2,3]. Genetic predisposition, overnutrition and certain dietary patterns like the so-called Western-style dietary pattern, as well as a lack of physical activity and changes in the intestinal microbiota and barrier function, are thought to be critical in the development of NAFLD [4,5,6,7,8]. However, the molecular mechanisms involved are still not fully understood and therapeutic options are mostly limited to lifestyle interventions [9].

Butyric acid is a short-chain fatty acid being built by microbial anaerobic fermentation of non-digestible polysaccharides. It is found in foods like milk and milk-products but also in the oral cavity and intestinal tract of humans and mammals [10]. Besides being a main energy source for colonocytes and intestinal epithelium, butyrate has also been shown to exhibit immunomodulatory and anti-inflammatory properties. Furthermore, the short-chain fatty acid has also various other biological effects such as the regulation of metabolism and maintenance of intestinal homeostasis [11]. In the early 1980s, it was reported that short-chain fatty acids possess therapeutic potential in some forms of colitis [12], which has since been in part confirmed in animal and human studies for Crohn’s disease. Recently, oral butyrate supplementation was reported to decrease cytokine release in patients with metabolic syndrome [13]. In addition, studies also suggest that an oral supplementation of therapeutic doses of sodium butyrate (SoB) (0.2–0.6 g/kg bw/d) may attenuate insulin resistance and the development of NAFLD, e.g., steatosis, inflammation and even early signs of fibrosis in rodents [6,14,15,16]. In these studies, it was shown that the beneficial effects of SoB on the development of NAFLD are associated with protection against the induction of inducible nitric oxide synthase (iNOS) and lipid peroxidation. Furthermore, the results of studies in FXR knockout mice suggest that the protective effects of the supplementation of SoB on the development of NAFLD may also, at least in part, be related to a reduction in hepatic bile acid [17]. The results of the study of Liang et al. further suggest that a probiotic mixture of Lactobacillus and Bifidobacterium might reduce adiposity and inflammation through butyrate production and G-protein-coupled receptor 109A-regulated signaling [18]. Studies also reported that the protective effects of SoB supplementation are associated with alterations in the intestinal microbiota and barrier function [15], while others report an induction of the peroxisome proliferator-activated receptor-α and, subsequently, β-oxidation in liver tissue [14], respectively. Others found no effects on intestinal tight junction protein, but rather an induction in intestinal melatonin synthesis and, subsequently, melatonin signaling in liver tissue [19]. However, despite intense research efforts and these more recent findings, the molecular mechanisms underlying the beneficial effects of oral SoB supplementation are still unclear. The present study aimed to determine whether an oral treatment with SoB protects mice with diet-induced early signs of NASH from disease progression, even in the absence of any change in diet, and to determine the mechanisms involved.

## 2. Materials and Methods

### 2.1. Animals and Treatments

**Figure 1:**
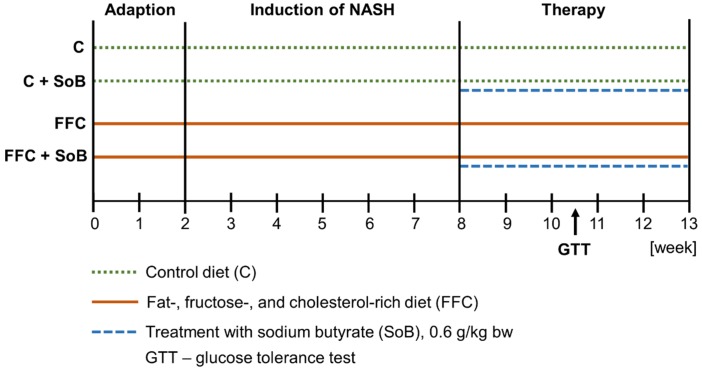
Study design and treatment groups. After an adaption phase during which mice were adapted to consuming a liquid diet, animals were either fed a C or an FFC diet. After 8 weeks, feeding of C and FFC was either sustained or animals were fed the different diets enriched with 0.6 g SoB/kg bw for 5 weeks. In week 11, all animals underwent a GTT. C, control diet; FFC, fat-, fructose-, and cholesterol-rich diet; GTT, glucose tolerance test; NASH, non-alcoholic steatohepatitis; SoB, sodium butyrate.

**Figure 2:**
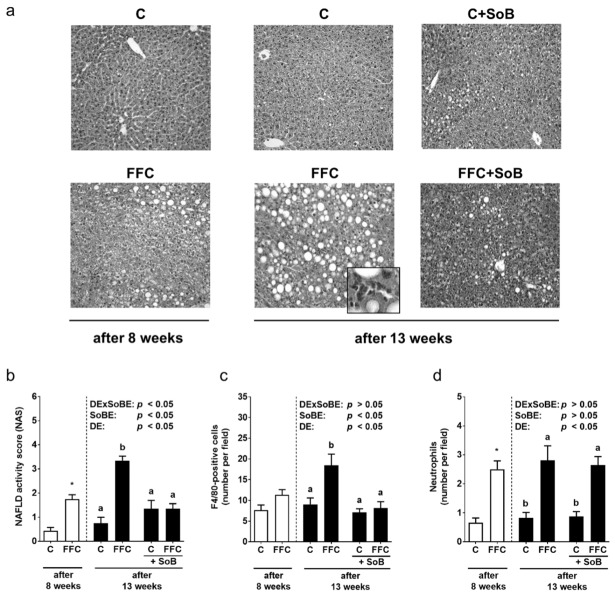
Effect of supplementation of SoB on liver status in mice with FFC-induced NASH. (**a**) Representative photomicrographs of hematoxylin and eosin staining of liver sections (magnification 200× and 400×), (**b**) evaluation of liver damage using a non-alcoholic fatty liver disease activity score (NAS), number of (**c**) F4/80-positive cells and (**d**) neutrophils per microscopic field in the livers. Data are expressed as means ± SEM, *n* = 8. Unpaired Student´s *t*-test was used to compare C and FFC after 8 weeks of feeding, * *p* < 0.05 compared with mice fed a C diet for 8 weeks. Two-way ANOVA was used to compare C, FFC, C+SoB and FFC+SoB after 13 weeks of feeding. Data with different letters are significantly different, *p* < 0.05. C, control diet; DE, diet effect; DExSoBE, interaction between diet and SoB; FFC, fat-, fructose-, and cholesterol-rich diet; NAFLD, non-alcoholic fatty liver disease; NASH, non-alcoholic steatohepatitis; SoB, sodium butyrate; SoBE, sodium butyrate effect.

Eight-week-old female C57BL/6J mice (Janvier SAS, Le-Genest-Saint-Isle, France) shown previously to be more susceptible to the development of fructose-induced steatosis [20] and to develop early signs of NASH at a similar rate as male mice [21], were housed in a specific-pathogen-free barrier facility accredited by the Association for Assessment and Accreditation of Laboratory Animal Care. Mice had free access to tap water at all times. All procedures were approved and registered by the local Institutional for Animal Care and Use Committee (Landesamt für Verbraucherschutz, reference number: 02-021/14, Thuringia, Germany). All animals were handled in accordance with the European Convention for the Protection of Vertebrate Animals used for Experimental and other Scientific Purposes. For the everted gut sac experiments detailed below, naïve mice without further treatments were killed by cervical dislocation. To induce early stages of NASH, mice were pair-fed a liquid fat-, fructose- and cholesterol-rich diet (FFC; 17.8 MJ/kg diet: 60E% from carbohydrates, 25E% from fat and 15E% from protein with 50% wt/wt fructose and 0.16% wt/wt cholesterol; Ssniff, Soest, Germany). Control animals were fed a standard liquid diet (C; 15.7 MJ/kg diet: 69E% from carbohydrates, 12E% from fat and 19E% from protein; Ssniff, Soest, Germany) as detailed previously [22]. For the pair-feeding of mice, liquid diet was administered in bottles with ball nipples. To achieve equal caloric intakes, the liquid diet intake of mice in each group was assessed daily and mean caloric intake per group per day was calculated. The amount of diet and calories in the different groups were then adjusted to the group with the lowest caloric intake at the next day, whereas the group with the lowest caloric intake was fed ad libitum (pair-feeding model) [22]. After 8 weeks of feeding, mice were assigned to the following groups (*n* = 8/group): C-fed, FFC-fed, C-fed mice receiving C supplemented with 0.6 g SoB/kg bw (C+SoB; Sigma-Aldrich, Steinheim, Germany) or FFC-fed mice receiving FFC supplemented with 0.6 g SoB/kg bw (FFC+SoB). SoB was supplemented for 5 weeks to the respective diets. This oral dose of SoB has been shown before to possess protective effects on the development of NAFLD in mice without any adverse side effects [6]. The study design is summarized in Figure 1. At week 11, mice were fasted for 6h followed by a glucose tolerance test (GTT) as detailed previously [22]. After 13 weeks, mice were anesthetized through the intraperitoneal injection of 100 mg ketamine + 16 mg xylazine/kg bw. Blood from the portal vein was collected just prior to sacrifice. Liver and intestinal tissue were fixed in neutral-buffered formalin or snap frozen in liquid nitrogen. For comparison of the effects of the different diets on the liver and body weight found after 8 weeks, data from a mouse experiment [22] run in parallel in the mouse facility in which mice were pair-fed C or FFC, were included in the present study (Table 1, Figure 2).

### 2.2. Everted Gut Sac Model of Mice

The small intestine (*n* = 4–6/treatment) was everted with a rod, as described by others [23] and cut into equal length sections. Each sac was ligated at both ends and filled with 1× Krebs–Henseleit-bicarbonate-buffer (KRH buffer). Scrapped mucosa and whole intestinal tissue from each sac were snap frozen for further analysis after being incubated in a gassed KRH buffer (95% O_2_/5% CO_2_), supplemented with 0, 3 and 6 mM SoB; 10 mM fructose or 6 mM nicotinamide adenine dinucleotide phosphate (NADPH, reduced form), respectively, at 37 °C for 1h.

### 2.3. Cell Culture

J774A.1 cells (DSMZ, Braunschweig, Germany) were cultured in DMEM (Pan Biotech, Germany) supplemented with 10% fetal bovine serum (Pan Biotech, Germany) and 1% penicillin and streptomycin at 37 °C in a humidified 5% CO_2_ atmosphere. At 80% confluence, cells were stimulated with 50 ng/mL lipopolysaccharide (LPS, Serotype: O55:B5, Sigma-Aldrich, Steinheim, Germany) with or without 0.6 mM SoB for 18h. The concentration of SoB was chosen based on a pilot experiment. Supernatant was collected and cells were lysed with peqGOLD Trifast (VWR, Germany) and stored at -80 °C for subsequent RNA isolation.

### 2.4. Histological Evaluation and Immunohistochemical Staining

Liver histology was assessed using the NAFLD activity score (NAS), as described previously [24]. Staining and counting of the number of neutrophilic granulocytes and assessment of hepatic fibrosis were carried out as described previously [25]. Liver sections were stained for F4/80, iNOS and 4-hydroxynonenal protein adducts (4-HNE) using polyclonal antibodies (F4/80: Abcam, Cambridge, UK; iNOS: Affinity BioReagents, Rockford, USA; 4-HNE: AG Scientific, San Diego, USA) and staining was evaluated as described before [6]. Paraffin-embedded sections of proximal small intestine (4 µm) were stained and analysed for the tight junction proteins occludin, zonula occludens 1 (ZO-1) and hydroxyindole-O-methyltransferase (HIOMT), respectively, using polyclonal primary antibodies (occludin and ZO-1: Invitrogen, CA, USA; HIOMT: Biozol Diagnostica GmbH, Germany) as previously described [19].

### 2.5. Blood Parameters of Liver Damage

The activities of alanine aminotransferase (ALT) and aspartate aminotransferase (AST) in plasma were determined using standard techniques in the routine laboratory of the University Hospital of Jena, Germany (Architect, Abbott, Wiesbaden, Germany).

### 2.6. Endotoxin Assay

Endotoxin levels were measured in portal plasma using a commercially available limulus amebocyte lysate assay (Charles River, France) as previously described [22]. Recovery rates were 90–124%.

### 2.7. Griess Assay

Nitric oxide (NO_2_^−^) concentrations in cell culture supernatant were measured with Griess reagent kit (Promega, Mannheim, Germany) according to the instructions of the manufacturers.

### 2.8. RNA Isolation and Real-Time RT-PCR

RNA isolation and real-time PCR were performed as detailed previously [6] using a SYBR Green^®^ Supermix (Agilent Technologies, Böblingen, Germany) and iTaq^TM^ Universal SYBR^®^ Green Supermix (Bio-Rad Ges.m.b.H., Vienna, Austria). Primer sequences are shown in Table S1. The number of targets was determined with the comparative cycle threshold (CT) method, which was normalized to an endogenous reference (18S) and relative to a calibrator (2^−ΔΔCt^).

### 2.9. ELISA and HDAC Enzymes Activity Assay

Hepatic tumor necrosis factor alpha (TNFα) and interleukin 6 (IL-6) protein concentrations as well as the melatonin and serotonin concentration in the proximal small intestine were determined using commercially available ELISA kits following the instructions of the manufacturers (TNFα: AssayPro, St. Charles, USA; IL-6: RayBiotech Inc, Norcross, USA; Melatonin and serotonin: IBL International GmbH, Hamburg, Germany). To determine histone deacetylase (HDAC) enzyme activity, nuclear proteins using a commercially available nuclear extraction kit (Active Motif, La Hulpe, Belgium) were isolated according to the manufacturer’s protocol. The measurement of HDAC activity was performed with the fluorescent HDAC Assay Kit (Active Motif, La Hulpe, Belgium) as described in the manual, with the following changes: per reaction, 5 µg nuclear extract of cells from intestinal tissue were used and the incubation time was extended to 1h 15 min to increase signal strength. All samples were measured both directly and in combination with the commercial HDAC inhibitor Trichostatin A (TSA; 1 µM) for the complete inhibition of all HDAC enzymes in the nuclear extracts to determine 0% HDAC activity as background for every sample.

### 2.10. Western Blot Analysis

To determine the protein levels of histone 3, cells from intestinal tissue were collected (approx. 1 × 10^7^ cells) and resuspended in 150 µl lysing-buffer (1% Nonident P40, 0.5 M Tris-Base (pH 7.6), 0.15 M NaCl, cOmplete^TM^ ULTRA Tablet/10 mL (Roche Diagnostics, Indianapolis, USA)). Lysates were stored at −80 °C, thawed and refrozen three times and treated with sonification. Cellular proteins were separated on 12% SDS-polyacrylamide gels and transferred to polyvinylidene difluoride membranes (Hybond-P, Amersham Biosciences, Piscataway, USA). Membranes were blocked in Tris-buffered saline (150 mmol/l NaCl, 13 mmol/l Tris, pH 7.5) containing 5% non-fat dry milk powder and were incubated with anti-β-actin (1:4,000, Sigma-Aldrich, Munich, Germany) or anti-acetyl-histone H3 (Lys9) (1:1,000, Cell Signaling Technology, Danvers, USA) overnight at 4 °C, and then incubated with peroxidase-conjugated anti-rabbit (1:5,000, Bio Rad, Hercules, USA) or anti-mouse (1:5,000, Bio Rad) for 45 min. Membranes were detected by the ECL Western blotting detection system on Hyperfilm-ECL (Amersham Biosciences). To determine the phosphorylation of AANAT and total AANAT, proteins were isolated with urea/DTT of scrapped mucosa obtained from everted gut sacs of naïve mice. Protein lysates (10 µg) were separated on a SDS-PAGE gel electrophoresis and transferred on a polyvinylidene difluoride membrane. Membranes were incubated with primary antibodies against pAANAT or AANAT (1:1,1000, biorbyt, Cambridge, UK, respectively) and secondary antibody (1:5,000, anti-rabbit, Cell Signaling Technology, Danvers, USA). Protein bands were detected with Super Signal West Dura Kit (Thermo Fisher Scientific, Waltham, MA, USA). Densitometric analysis were performed using ChemiDoc XRS System.

### 2.11. Statistical Analysis

All results are shown as means ± standard error of mean (SEM). To identify outliers, Grubb’s test was used. Bartlett’s test was used to determine the homogeneity of variances, and log-transformation of values was performed when values were not normally distributed. Unpaired Student´s *t*-test was used to determine statistically significant differences between parameters assessed in mice fed C or FFC for 8 weeks or where applicable. One- and two-way ANOVA with Tukey’s post hoc test were applied to determine statistical differences between groups, as indicated (Graph Pad Prism, Version 6.0, San Diego, CA, USA). *p* value < 0.05 was considered to be significant.

## 3. Results

### 3.1. Body Weight and Markers of Liver Damage

While caloric intake and weight gain were similar between C- and FFC-fed mice, FFC-fed mice had developed steatosis with early signs of inflammation after 8 weeks of feeding. Indeed, NAFLD activity score (NAS), absolute liver weight and liver to body weight ratio were significantly higher in FFC-fed mice than in controls (C vs. FFC, *p* < 0.05 for all parameters; Table 1, Figure 2). The number of F4/80-positive cells in the liver as well as AST and ALT activity in plasma were similar between C- and FFC-fed mice after 8 weeks of feeding. Despite having a similar caloric intake, the absolute body weight gain of FFC-fed mice was significantly higher than C-fed mice after 13 weeks of feeding. The signs of NAFLD had progressed to early steatohepatitis. Indeed, total NAS, the numbers of F4/80-positive cells and neutrophils in liver tissue, as well as ALT and AST activities in plasma, were significantly higher than in controls. In contrast, in FFC+SoB-fed mice, the NAS and the number of F4/80-positive cells were significantly lower than in FFC-fed mice, with neither parameter differing from either control group (Figure 2). ALT and AST activity in plasma, as well as the number of neutrophils in liver tissue, were significantly higher in FFC+SoB-fed when compared with control animals and did not differ from FFC-fed animals (Table 1, Figure 2).

In line with the findings for inflammation and F4/80-positive cells, the protein levels of TNFα and IL-6 in the liver tissue were significantly higher in the livers of FFC-fed mice when compared to all other groups (Table 1). The protein levels of TNFα and IL-6 in the liver tissue of FFC+SoB-fed mice were similar to controls. After 13 weeks of feeding, neither FFC-fed nor FFC+SoB-fed mice displayed any signs of liver fibrosis, as determined by sirius red staining and mRNA expression of alpha smooth muscle actin (*αSma*) and transforming growth factor beta (*Tgfβ*) (Table S2).

### 3.2. Parameters of Glucose Metabolism

**Figure 3:**
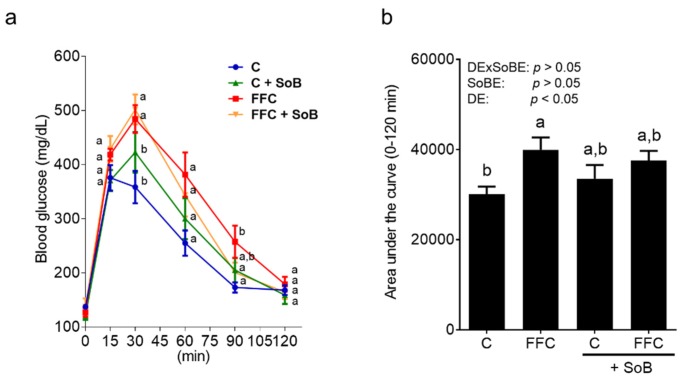
Effect of supplementation of SoB on glucose metabolism in mice with FFC-induced NASH. (**a**) Blood glucose levels during glucose tolerance test (GTT) and, (**b**) quantitative analysis of area under the curve of GTT (0-120 min). Data are expressed as means ± SEM, *n* = 8. Data with different letters are significantly different, *p* < 0.05. C, control diet; DE, diet effect; DExSoBE, interaction between diet and SoB; FFC, fat-, fructose-, and cholesterol-rich diet; NASH, non-alcoholic steatohepatitis; SoB, sodium butyrate; SoBE; sodium butyrate effect.

Fasting blood glucose levels were similar between groups. Thirty minutes after the glucose challenge, blood glucose levels in both FFC-fed groups regardless of additional treatments were significantly higher than in controls (Figure 3). However, 90 min after the glucose injection, only the blood glucose levels of FFC-fed mice were significantly higher than those of controls, while blood glucose levels in FFC+SoB-fed mice were at the level of controls (Figure 3). The area under the curve (AUC) of GTT was also significantly higher in FFC-fed mice when compared to controls, whereas the AUC of FFC+SoB-fed mice was similar to both control groups (Figure 3).

### 3.3. Markers of Lipid Peroxidation

**Figure 4:**
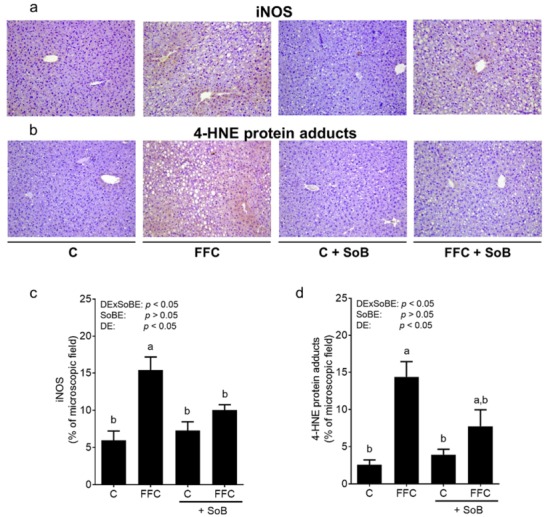
Effect of supplementation of SoB on lipid peroxidation in livers of mice with FFC-induced NASH. Representative photomicrographs of (**a**) inducible nitric oxide synthase (iNOS) and (**b**) 4-hydroxynonenal (4-HNE) protein adducts staining in paraffin embedded tissue (magnification 200×) as well as densitometric analysis of (**c**) iNOS and (**d**) 4-HNE protein adducts staining in liver tissue. Data are expressed as means ± SEM, *n* = 8. Data with different letters are significantly different, *p* < 0.05. C, control diet; DE, diet effect; DExSoBE, interaction between diet and SoB; FFC, fat-, fructose-, and cholesterol-rich diet; NASH, non-alcoholic steatohepatitis; SoB, sodium butyrate; SoBE; sodium butyrate effect.

To further delineate the molecular mechanisms underlying the beneficial effects of SoB, we next determined the concentrations of iNOS protein and 4-HNE protein adducts that were significantly higher in the livers of FFC-fed mice than in both control groups. Both parameters were almost at the level of controls in livers of FFC+SoB-fed mice (Figure 4). To determine if SoB directly effects lipopolysaccharide (LPS)- and Tlr4-dependent signaling cascades, J774A.1 cells, described as a model of Kupffer cells, were challenged with LPS in the presence or absence of 0.6 mM SoB for 18h. As expected, the NO_2_^−^ concentration, mRNA expressions of *iNos*, *Tnfα*, *Il1β* and *Il6* were significantly induced in LPS-stimulated cells. The addition of 0.6 mM SoB had no effect on the LPS-dependent induction of any of these parameters (Table S3).

### 3.4. Tight Junction Proteins, Portal Endotoxin and Tlr4-Dependent Signaling Pathway

**Figure 5:**
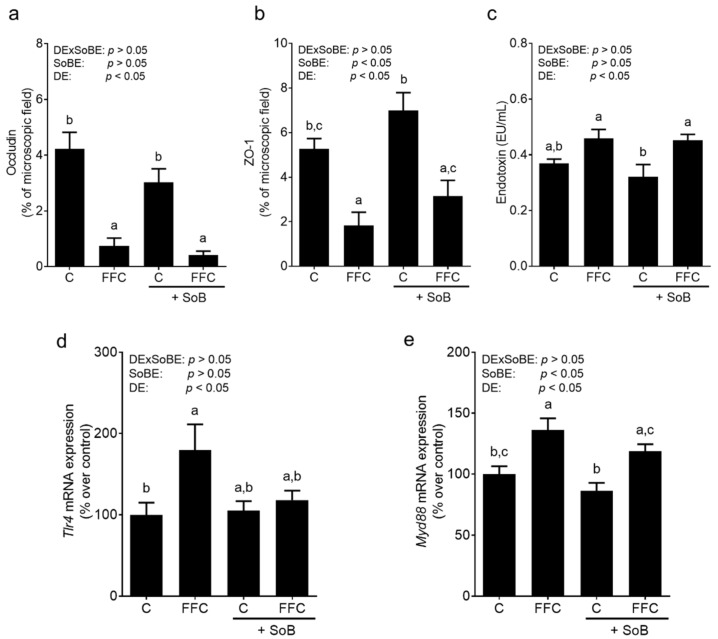
Effect of supplementation of SoB on tight junction proteins in upper parts of the small intestine, bacterial endotoxin levels and on markers of the toll-like receptor 4 (Tlr4) signaling cascade of mice with FFC-induced NASH. Qualitative analysis of (**a**) occludin, (**b**) ZO-1 protein staining in proximal small intestine, (**c**) bacterial endotoxin concentration in portal plasma as well as expression of (**d**) *Tlr4* and (**e**) myeloid differentiation primary response gene 88 (*Myd88*) mRNA in liver tissue. Data are expressed as means ± SEM, *n* = 6–8. Data with different letters are significantly different, *p* < 0.05. C, control diet; DE, diet effect; DExSoBE, interaction between diet and SoB; FFC, fat-, fructose-, and cholesterol-rich diet; NASH, non-alcoholic steatohepatitis; SoB, sodium butyrate; SoBE; sodium butyrate effect, ZO-1, zona occludens 1.

As a loss of tight junction proteins and an increased translocation of bacterial endotoxin has been shown to be involved in the induction of iNOS and increased formation of reactive oxygen species in liver tissue in settings of NAFLD [26], we next determined the markers of intestinal barrier function. The protein levels of occludin and ZO-1 in upper parts of the small intestine were lower in both FFC-fed groups when compared with control groups (occludin: *p* < 0.05 for FFC groups vs. C groups; ZO-1: *p* < 0.05 for C vs. FFC and C+SoB vs. both FFC groups; Figure 5). No differences were found between FFC-fed groups. Representative pictures of staining of occludin and ZO-1 are shown in Figure S1. Bacterial endotoxin concentrations in the portal plasma were significantly and by trend (*p* = 0.14 compared to C) higher in both FFC-fed groups than in controls, respectively, while concentrations were similar between FFC- and FFC+SoB-fed mice (Figure 5).

### 3.5. Intestinal Melatonin Metabolism and Melatonin Receptors in Liver Tissue

**Figure 6:**
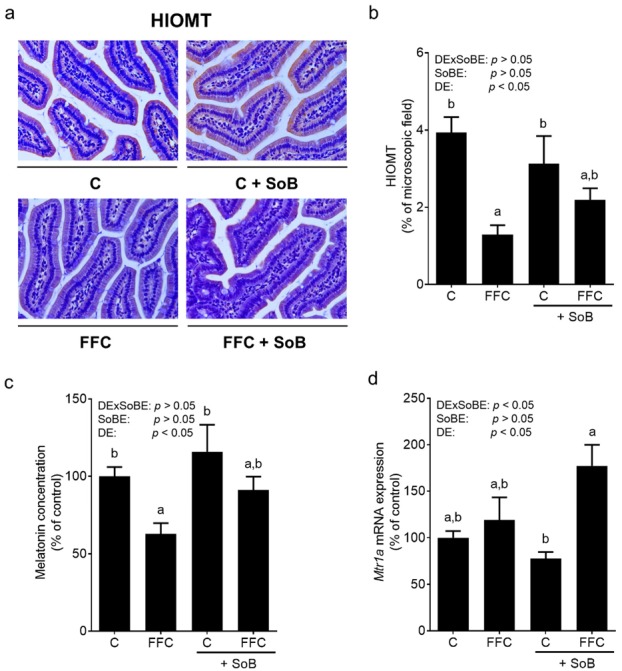
Effect of supplementation of SoB on enzymes involved in melatonin synthesis and expression of melatonin receptor in livers of mice with FFC-induced NASH. (**a**) Representative photomicrographs of HIOMT staining, (**b**) densitometric analysis of HIOMT protein concentration, (**c**) melatonin concentration in the upper part of the small intestine and (**d**) mRNA expression of *Mtr1a* in liver tissue. Data are expressed as means ± SEM, *n* = 8. Data with different letters are significantly different, *p* < 0.05. C, control diet; DE, diet effect; DExSoBE, interaction between diet and SoB; FFC, fat-, fructose-, and cholesterol-rich diet; HIOMT, hydroxyindole-O-methyltransferase; Mtr1a, melatonin receptor 1a; NASH, non-alcoholic steatohepatitis; SoB, sodium butyrate; SoBE, sodium butyrate effect.

Protein levels of HIOMT, a key enzyme of melatonin synthesis [27], were significantly lower in the small intestine of FFC-fed mice compared to both control groups. In contrast, in the small intestine of FFC+SoB-fed mice HIOMT, protein concentration was almost at the level of control groups (Figure 6). Melatonin concentration in the small intestine was also significantly lower in FFC-fed mice than in both control groups. Similar differences were not found between FFC+SoB-fed mice and both control groups. The expression of melatonin receptor 1a (*Mtr1a*) mRNA in the livers of FFC+SoB-fed mice was significantly higher compared to C+SoB-fed mice while being similar between both control groups and FFC-fed mice (Figure 6). The expression of *Mtr1b* mRNA was not detectable in liver samples of mice.

### 3.6. Melatonin Metabolism and Activity of Histone Deacetylases (HDAC) Enzymes in Small Intestinal Tissue: Ex Vivo Experiments Using an Everted Gut Sac Model

**Figure 7:**
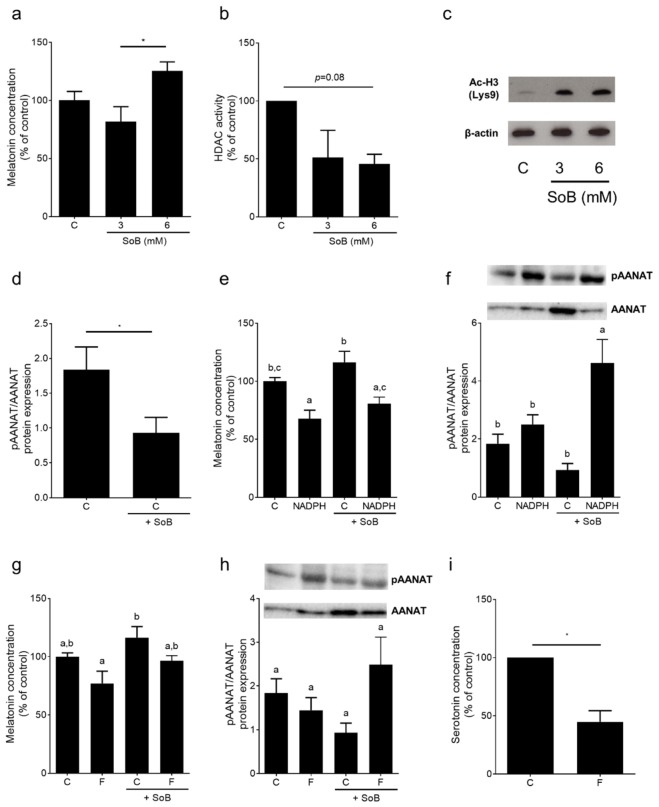
Effect of SoB on melatonin and serotonin concentration, protein expression of AANAT and activity of histone deacetylases enzymes in everted small intestinal sacs. (**a**) Melatonin concentration in whole intestinal tissue specimen, (**b**) activity of HDAC enzymes, and (**c**) representative blot of acetylated histone complex H3 protein expression treated with SoB. (**d**) Phosphorylated AANAT in mucosa of intestinal tissue obtained of everted sacs of naïve mice challenged with 6 mM SoB, (**e**,**g**) melatonin concentration and (**f**,**h**) phosphorylated AANAT in whole intestinal tissue obtained of everted sacs treated with 6 mM NADPH and/or 6 mM SoB as well as 10 mM fructose and/or 6 mM SoB. (**i**) Serotonin concentration in whole intestinal tissue specimen treated with 10 mM fructose. Data are expressed as means ± SEM, *n* = 3–6. * *p* < 0.05. Data with different letters are significantly different, *p* < 0.05. Aanat, serotonin N-acetyltransferase; Ac-H3 (Lys9), acetylated histone complex H3; C, everted gut sacs incubated only in 1 × Krebs–Henseleit-bicarbonate-buffer; F, fructose; HDAC, histone deacetylases; NADPH, nicotinamide adenine dinucleotide phosphate (reduced form); SoB, sodium butyrate.

As shown in Figure 7, melatonin concentration was significantly higher in a whole-tissue specimen obtained from the everted gut sacs of naïve mice treated with 6 mM SoB for 1h compared to those treated with 0 and 3 mM SoB (*p* < 0.05). The activity of HDAC enzymes was inhibited by ~50% in everted sacs challenged with 3 and 6 mM SoB, respectively, when compared to those without SoB treatment. The inhibition of HDAC enzymes was associated with the induction of acetylated histone complex H3. While the expression of serotonin N-acetyltransferase (*Aanat*) mRNA was similar between groups (Table S4), pAANAT levels were significantly lower in tissues treated with 6 mM SoB (Figure 7). *Mtr1a* mRNA expressions were similar between groups, whereas expressions of *Hiomt* and *Mtr1b* mRNA were below the level of detection (Table S4). As 6 mM SoB was found to exert the largest effects on melatonin levels, this concentration was employed in all further experiments. To further delineate the role of HDACs in the regulation of melatonin synthesis in the gut, everted sacs were treated with NADPH, which has been suggested to induce HDAC enzymes activity [28]. Melatonin concentration was significantly lower in tissue exposed to NADPH when compared to controls, an effect also found when tissue was incubated with both SoB and NADPH. The phosphorylation of Thr29 of AANAT, which has been suggested to lead to an inactivation of AANAT via proteasomal degradation [29], was super-induced in tissues treated with NADPH+SoB. In contrast, in all other groups, phosphorylation levels were at the level of controls. To further delineate the effects of fructose on intestinal melatonin synthesis, everted sacs were challenged with fructose in the presence and absence of SoB. In everted sacs challenged with 10 mM fructose, melatonin concentration was significantly lower than in those treated with SoB. While not altered in fructose-challenged tissue, pAANAT was higher in F+SoB-treated tissue when compared to SoB-treated tissue. Furthermore, the incubation of everted sacs with fructose was associated with a decrease in serotonin levels (~50%, *p* < 0.05) in small intestinal tissue (Figure 7).

## 4. Discussion

Despite intense research efforts and many novel therapeutic approaches, there is still no universally accepted therapy for the treatment of NAFLD other than lifestyle interventions. Here, we determine the therapeutic effects of an oral supplementation of SoB on a pre-existing hepatic steatosis. From weeks 8 to 13, disease progressed in FFC-fed animals from simple steatosis with slight signs of inflammation to macrovesicular steatosis with an increase in the number of inflammatory foci also associated with elevated levels of proinflammatory cytokines like TNFα and IL6. The oral supplementation of SoB significantly attenuated this progression in FFC-fed animals despite no changes in diet. Still, even after 13 weeks of feeding, signs of fibrosis were very limited in either FFC-fed group. This is in line with the earlier findings of our own group [22] and those of others [21] using similar diets. Somewhat contrasting these findings, the numbers of neutrophils were only slightly different between the two FFC-fed groups. It has been shown before that an increased TNFα production by resident macrophages is crucial in the early phase of NASH for the recruitment of blood-derived monocytes to the liver [30]. For the recruitment of neutrophils, other factors like an induction of lipocalin 2 [31] and Tlr9 [32] may be critical. The activities of AST and ALT in plasma were markedly higher in the FFC-fed group, further suggesting that SoB only partially “cured” the disease. Indeed, while liver histology was not significantly different between control groups and FFC+SoB-fed animals, there were still some signs of steatosis and inflammation present in FFC+SoB-fed mice.

The oral supplementation of SoB was also associated with the improved glucose tolerance of FFC-fed mice. These findings are in line with previous findings of our own [19] and other groups indicating that an oral SoB supplementation protects male rodents from the development of insulin resistance and impairments of glucose tolerance [33]. Indeed, the results of several studies suggest that SoB may modulate pancreatic β-cell function [34,35]. Taken together, our data suggest that orally supplemented SoB attenuates the progression of steatosis to steatohepatitis and the development of insulin resistance thereby further bolsters the previous findings of us and others [6,15,19]. However, if an oral supplementation of SoB also attenuates the development of diet-induced later phases of hepatic fibrosis, as well as if similar beneficial effects are also found in male mice, remains to be determined.

The induction of iNOS and increased lipid peroxidation in liver have repeatedly been shown to be associated with the development of NAFLD, both in humans with NAFLD [36] and animal models of the disease [26]. Furthermore, the therapeutic effects of SoB have been associated with a reduction in the iNOS protein and markers of lipid peroxidation in previous studies of our own group [6] and those of others [37]. Indeed, the induction of iNOS has been shown before to be triggered through LPS-Tlr4-dependent signaling pathways [26]. In the present study, the induction of iNOS and increase in the 4-HNE protein adduct concentration were almost completely attenuated in FFC-fed mice treated with SoB. However, the results of our in vitro studies employing J774A.1 cells, as a model of Kupffer cells, suggest that SoB has no, or very limited, direct effects on the LPS-induced activation of Kupffer cells. Indeed, our results suggest that SoB may affect the development of NAFLD through indirect mechanisms (see below).

Elevated bacterial endotoxin levels and, subsequently, the activation of Tlr4-dependent signaling cascades in liver are believed to be among the key risk factors for the development of NAFLD (for overview, see [7]). The results of studies aiming to modulate the intestinal bioavailability of butyrate and other short-chain fatty acids through targeting intestinal microbiota or supplementing SoB suggest that the beneficial effects may result from an improved intestinal barrier function and lower translocation of bacterial endotoxin (for overview, see [38] and [15,39]). Indeed, in the present study, while the mRNA expression of *Tlr4* in liver tissue was only significantly higher in FFC-fed animals, *Myd88* mRNA expression was induced in the livers of both FFC-fed groups. Myd88 is not only an adaptor protein of Tlr4 but is also involved in the signaling of other Tlrs (for overview, see [40]), several of which have been shown to be induced in patients and animals with NAFLD [36,41]. Myd88 is not solely regulated at the level of expression [42]. Somewhat contrasting the findings for *Tlr4* mRNA expression in liver tissue, increases in bacterial endotoxin levels in portal blood and the loss of protein levels of tight junction proteins in the proximal small intestine were similar between the two FFC-fed groups, regardless of additional treatments. While these findings are in line with the previous studies of our own group, employing similar SoB doses but different feeding models [6,19], these data are in contrast to the earlier findings of other groups. Indeed, it was reported by others [15,34] that the protective effects of SoB on the development of NAFLD in mice treated orally with SoB are associated with protection against impairments in intestinal barrier function, e.g., the loss of the tight junction protein and increases in bacterial endotoxin levels in serum. Furthermore, it was reported that these beneficial effects of SoB in the settings of diet-induced NAFLD in animal models were associated with marked changes in microbiota composition [15,16]. Differences between the present study and those of others might have resulted from differences in study design, e.g., differences in the composition of diet, the concentration of SoB, length of treatment and gender of animals. Indeed, it has been suggested before that male and female mice markedly differ with regards to their susceptibility to the loss of tight junction proteins and intestinal permeability when exposed to alcohol or a fructose-rich diet, with female animals being more sensitive [20,43]. Accordingly, it could be that the dose of SoB used in the present study was not sufficient to “restore” intestinal barrier function in female mice. Due to a lack of samples, it was not possible to determine intestinal microbiota composition. When interpreting the results of the present study, it has to be acknowledge that the macronutrient composition of C and FFC diet differed not only in fat and carbohydrates, but also in protein content (C diet 19E% protein and FFC diet 15E% protein). The impact of all these factors on experimental outcome needs to be addressed in future studies.

The results of in vitro and in vivo studies suggest that SoB can modulate the expression of enzymes involved in the synthesis of melatonin, e.g., AANAT and HIOMT [19,44,45], and may even lead to an elevation in melatonin concentration in the small intestinal tissue as well as *Mtr1a* expression in the liver of mice with fructose-induced NAFLD [19]. Indeed, it has been reported that treatment with melatonin and its precursor tryptophan, respectively, attenuates signs of diet-induced NAFLD in rodents [46,47] and may reduce the plasma levels of proinflammatory cytokines in NAFLD patients [48]. Here, the concentrations of HIOMT protein and melatonin were lower in the small intestine of FFC-fed mice, while in FFC-fed mice treated for 5 weeks with SoB, the concentrations were at the level of controls. Furthermore, the expression of *Mtr1a*, shown before to be highly dependent upon the presence of melatonin [19], was also significantly higher in the livers of FFC+SoB-fed mice, but not in those of FFC-fed mice. The results of studies of others also suggest that melatonin may decrease *Tlr4* expression, and subsequently the induction of dependent signaling cascades including NFκB and iNOS, and that these changes in *Tlr4* expression may be independent of Myd88 [49,50,51]. Supporting the hypothesis that SoB alters melatonin synthesis in the small intestine, the incubation of everted sacs with SoB was associated with an increase in melatonin levels and a decrease in pAANAT (Thr29), the latter suggested to play a role in the inactivation of AANAT through proteasomal degradation [29]. These effects on melatonin concentration and pAANAT were related to an inhibition of HDAC activity and were abolished when an activator of HDAC activity e.g., NADPH, or fructose, were present. HDAC activity has been shown before to be inhibited by SoB [28,52]. These data suggest that the changes in melatonin in the small intestine found in our present study might have resulted from the modulation of HDAC activity and inactivation/degradation of AANAT. Furthermore, the results of others and our own group suggest that fructose may decrease serotonin bioavailability through a decrease in serotonin and SERT-mediated reuptake [53,54]. Indeed, here we showed that, after being exposed to fructose for only 1h, serotonin levels were significantly decreased in the small intestine. However, the underlying mechanisms need to be delineated in future studies.

## 5. Conclusions

In summary, the results of the present study suggest that an oral supplementation of SoB at pharmacological doses prevents the progression of a pre-existing steatosis, beginning inflammation in mice even when an NAFLD-inducing dietary pattern is continued. Our data further bolster the hypothesis that the beneficial effects of an oral SoB supplementation on diet-induced NAFLD results in protection against the induction of iNOS and lipid peroxidation in the liver. Our data further suggest that this, at least in part, may result from the induction of intestinal melatonin synthesis and dependent signaling cascades in liver. However, further studies are needed to fully unravel the molecular mechanisms involved. Although SoB is already commercially available and a prescription is not required, the effectiveness of SoB formulations (e.g., powder, capsules) as well as the doses necessary to achieve an effect in patients with NAFLD, need to be assessed in future studies, as the SoB doses (0.6 g/kg bw) used in the present study in animals, but also in ex vivo experiments, were markedly higher than those used in human studies. Indeed, the oral doses of SoB applied to humans to assess the effects of SoB on diseases of other etiologies, e.g., metabolic syndrome or diabetes mellitus type 2, ranged from 0.6 to 4 g/d [55,56].

## Figures and Tables

**Table 1:** Effect of an oral supplementation of SoB on caloric intake, body- and liver weight as well as parameters of liver damage in mice with FFC-induced NASH.

Diet Groups									
	0–8 Weeks		8–13 Weeks				*p* (Two-Way ANOVA)		
	C	FFC	C	FFC	C+SoB	FFC+SoB	DE × SoBE	SoBE	DE
**Caloric intake (kcal/g bw/d)**	0.49 ± 0.01	0.49 ± 0.01	0.47 ± 0.01 ^a^	0.49 ± 0.01 ^a^	0.47 ± 0.01 ^a^	0.48 ± 0.01 ^a^	>0.05	>0.05	>0.05
**Absolute body weight gain (g)**	2.9 ± 0.2	3.6 ± 0.4	4.3 0.4 ^a,b^	5.8 ± 0.4 ^a^	4.5 ± 0.6 ^b^	5.3 ± 0.4 ^a,b^	>0.05	>0.05	<0.05
**Absolute body weight (g)**	20 ± 0.4	22 ± 0.2 *	22.3 ± 0.5 ^a^	23.4 ± 0.5 ^a^	22.4 ± 0.3 ^a^	23.3 ± 0.6 ^a^	>0.05	>0.05	>0.05
**Liver weight (g)**	0.9 ± 0	1.4 ± 0 *	1 ± 0.1 ^b^	1.5 ± 0 ^a^	1.1 ± 0 ^b^	1.4 ± 0.1 ^a^	<0.05	>0.05	<0.05
**Liver/body weight ratio (%)**	4.5 ± 0.1	6.2 ± 0.1 *	4.7 ± 0.2 ^b^	6.3 ± 0.2 ^a^	5 ± 0.2 ^b^	6.2 ± 0.2 ^a^	>0.05	>0.05	<0.05
**ALT (U/L)**	22.5 ± 2.5	38.5 8.7	12.8 0.5 ^b^	34.9 ± 9.1 ^a^	14.5 ± 1.7 ^b^	40.4 ± 5 ^a^	>0.05	>0.05	<0.05
**AST (U/L)**	49.8 ± 4.6	68.4 ± 11.3	35.4 ± 1.4 ^b^	82.4 ± 19.1 ^a^	38.8 ± 3.6 ^b^	81.2 ± 9.8 ^a^	>0.05	>0.05	<0.05
**TNFα (pg/mg protein)**	n.d.	n.d.	26.7 ± 0.9 ^b^	44.4 ± 5.6 ^a^	26.2 ± 2.2 ^b^	28.4 ± 1.1 ^b^	<0.05	<0.05	<0.05
**IL-6 (pg/mg protein)**	n.d.	n.d.	96.7 ± 5.3 ^b^	135.3 ± 4.1 ^a^	99.7 ± 4.3 ^b^	107.6 ± 4.4 ^b^	<0.05	<0.05	<0.05

Data are shown as means ± SEM, *n* = 8. Unpaired Student´s *t*-test was used to compare C and FFC after 8 weeks of feeding (0–8 weeks), * *p* < 0.05 compared with mice fed a C diet for 8 weeks. Two-way analysis of variance (ANOVA) was used to compare C, FFC, C+SoB and FFC+SoB after 13 weeks of feeding (8–13 weeks). Data with different letters are significantly different, *p* < 0.05. ALT, alanine aminotransferase; AST, aspartate aminotransferase; C, control diet; DE, diet effect; DExSoBE, interaction between diet and SoB; FFC, fat-, fructose-, and cholesterol-rich diet; IL, interleukin; NASH, non-alcoholic steatohepatitis; n.d., not detected; SoB, sodium butyrate; SoBE, sodium butyrate effect; TNFα, tumor necrosis factor alpha.

## Supplementary Materials

The following are available online at https://www.mdpi.com/2072-6643/12/4/951/s1, Figure S1: Effect of supplementation of sodium butyrate on tight junction proteins in upper parts of the small intestine of mice with FFC-induced NASH, Table S1: Primer sequences used for real-time PCR, Table S2: Effect of an oral supplementation of SoB on fibrosis markers in livers of mice with FFC-induced NASH, Table S3: Effect of SoB on NO_2_^−^ concentration as well as mRNA expression of *iNos* and proinflammatory mediators in J774A.1 cells, Table S4: Effect of SoB on mRNA expression of melatonin receptor, Aanat and Hiomt in small intestinal tissue of an everted gut sac model.

## References

[1] Younossi Z.M., Koenig A.B., Abdelatif D., Fazel Y., Henry L., Wymer M. (2016). Global epidemiology of nonalcoholic fatty liver disease-meta-analytic assessment of prevalence, incidence, and outcomes. Hepatology.

[2] Sass D.A., Chang P., Chopra K.B. (2005). Nonalcoholic fatty liver disease: A clinical review. Dig. Dis. Sci..

[3] Neuschwander-Tetri B.A., Caldwell S.H. (2003). Nonalcoholic steatohepatitis: Summary of an aasld single topic conference. Hepatology.

[4] Tsunoda K., Kai Y., Kitano N., Uchida K., Kuchiki T., Nagamatsu T. (2016). Impact of physical activity on nonalcoholic steatohepatitis in people with nonalcoholic simple fatty liver: A prospective cohort study. Prev. Med..

[5] Edmison J., McCullough A.J. (2007). Pathogenesis of non-alcoholic steatohepatitis: Human data. Clin. Liver Dis..

[6] Jin C.J., Sellmann C., Engstler A.J., Ziegenhardt D., Bergheim I. (2015). Supplementation of sodium butyrate protects mice from the development of non-alcoholic steatohepatitis (nash). Br. J. Nutr..

[7] Brandl K., Schnabl B. (2017). Intestinal microbiota and nonalcoholic steatohepatitis. Curr. Opin. Gastroenterol..

[8] Kirpich I.A., Marsano L.S., McClain C.J. (2015). Gut-liver axis, nutrition, and non-alcoholic fatty liver disease. Clin. Biochem..

[9] Zhu J.Z., Hollis-Hansen K., Wan X.Y., Fei S.J., Pang X.L., Meng F.D., Yu C.H., Li Y.M. (2016). Clinical guidelines of non-alcoholic fatty liver disease: A systematic review. World J. Gastroenterol..

[10] Canani R.B., Costanzo M.D., Leone L., Pedata M., Meli R., Calignano A. (2011). Potential beneficial effects of butyrate in intestinal and extraintestinal diseases. World J. Gastroenterol..

[11] Zhou D., Fan J.G. (2019). Microbial metabolites in non-alcoholic fatty liver disease. World J. Gastroenterol..

[12] Harig J.M., Soergel K.H., Komorowski R.A., Wood C.M. (1989). Treatment of diversion colitis with short-chain-fatty acid irrigation. N. Engl. J. Med..

[13] Cleophas M.C.P., Ratter J.M., Bekkering S., Quintin J., Schraa K., Stroes E.S., Netea M.G., Joosten L.A.B. (2019). Effects of oral butyrate supplementation on inflammatory potential of circulating peripheral blood mononuclear cells in healthy and obese males. Sci. Rep..

[14] Sun B., Jia Y., Hong J., Sun Q., Gao S., Hu Y., Zhao N., Zhao R. (2018). Sodium butyrate ameliorates high-fat-diet-induced non-alcoholic fatty liver disease through peroxisome proliferator-activated receptor alpha-mediated activation of beta oxidation and suppression of inflammation. J. Agric. Food Chem..

[15] Zhou D., Pan Q., Xin F.Z., Zhang R.N., He C.X., Chen G.Y., Liu C., Chen Y.W., Fan J.G. (2017). Sodium butyrate attenuates high-fat diet-induced steatohepatitis in mice by improving gut microbiota and gastrointestinal barrier. World J. Gastroenterol..

[16] Ye J., Lv L., Wu W., Li Y., Shi D., Fang D., Guo F., Jiang H., Yan R., Ye W. (2018). Butyrate protects mice against methionine-choline-deficient diet-induced non-alcoholic steatohepatitis by improving gut barrier function, attenuating inflammation and reducing endotoxin levels. Front. Microbiol..

[17] Sheng L., Jena P.K., Hu Y., Liu H.X., Nagar N., Kalanetra K.M., French S.W., French S.W., Mills D.A., Wan Y.Y. (2017). Hepatic inflammation caused by dysregulated bile acid synthesis is reversible by butyrate supplementation. J. Pathol..

[18] Liang Y., Lin C., Zhang Y., Deng Y., Liu C., Yang Q. (2018). Probiotic mixture of lactobacillus and bifidobacterium alleviates systemic adiposity and inflammation in non-alcoholic fatty liver disease rats through gpr109a and the commensal metabolite butyrate. Inflammopharmacology.

[19] Jin C.J., Engstler A.J., Sellmann C., Ziegenhardt D., Landmann M., Kanuri G., Lounis H., Schroder M., Vetter W., Bergheim I. (2016). Sodium butyrate protects mice from the development of the early signs of non-alcoholic fatty liver disease: Role of melatonin and lipid peroxidation. Br. J. Nutr..

[20] Spruss A., Henkel J., Kanuri G., Blank D., Puschel G.P., Bischoff S.C., Bergheim I. (2012). Female mice are more susceptible to nonalcoholic fatty liver disease: Sex-specific regulation of the hepatic amp-activated protein kinase-plasminogen activator inhibitor 1 cascade, but not the hepatic endotoxin response. Mol. Med..

[21] Marin V., Rosso N., Dal Ben M., Raseni A., Boschelle M., Degrassi C., Nemeckova I., Nachtigal P., Avellini C., Tiribelli C. (2016). An animal model for the juvenile non-alcoholic fatty liver disease and non-alcoholic steatohepatitis. PLoS ONE.

[22] Sellmann C., Baumann A., Brandt A., Jin C.J., Nier A., Bergheim I. (2017). Oral supplementation of glutamine attenuates the progression of nonalcoholic steatohepatitis in c57bl/6j mice. J. Nutr..

[23] Hamilton K.L., Butt A.G. (2013). Glucose transport into everted sacs of the small intestine of mice. Adv. Physiol. Educ..

[24] Sellmann C., Priebs J., Landmann M., Degen C., Engstler A.J., Jin C.J., Garttner S., Spruss A., Huber O., Bergheim I. (2015). Diets rich in fructose, fat or fructose and fat alter intestinal barrier function and lead to the development of nonalcoholic fatty liver disease over time. J. Nutr. Biochem..

[25] Bergheim I., Guo L., Davis M.A., Duveau I., Arteel G.E. (2006). Critical role of plasminogen activator inhibitor-1 in cholestatic liver injury and fibrosis. J. Pharmacol. Exp. Ther..

[26] Spruss A., Kanuri G., Uebel K., Bischoff S.C., Bergheim I. (2011). Role of the inducible nitric oxide synthase in the onset of fructose-induced steatosis in mice. Antioxid. Redox. Signal..

[27] Konturek S.J., Konturek P.C., Brzozowski T., Bubenik G.A. (2007). Role of melatonin in upper gastrointestinal tract. J. Physiol. Pharmacol..

[28] Vogelauer M., Krall A.S., McBrian M.A., Li J.Y., Kurdistani S.K. (2012). Stimulation of histone deacetylase activity by metabolites of intermediary metabolism. J. Biol. Chem..

[29] Vriend J., Liu W., Reiter R.J. (2017). The pineal gland: A model for adrenergic modulation of ubiquitin ligases. PLoS ONE.

[30] Tosello-Trampont A.C., Landes S.G., Nguyen V., Novobrantseva T.I., Hahn Y.S. (2012). Kuppfer cells trigger nonalcoholic steatohepatitis development in diet-induced mouse model through tumor necrosis factor-alpha production. J. Biol. Chem..

[31] Ye D., Yang K., Zang S., Lin Z., Chau H.T., Wang Y., Zhang J., Shi J., Xu A., Lin S. (2016). Lipocalin-2 mediates non-alcoholic steatohepatitis by promoting neutrophil-macrophage crosstalk via the induction of cxcr2. J. Hepatol..

[32] Mridha A.R., Haczeyni F., Yeh M.M., Haigh W.G., Ioannou G.N., Barn V., Ajamieh H., Adams L., Hamdorf J.M., Teoh N.C. (2017). Tlr9 is up-regulated in human and murine nash: Pivotal role in inflammatory recruitment and cell survival. Clin. Sci. (Lond.).

[33] Aguilar E.C., da Silva J.F., Navia-Pelaez J.M., Leonel A.J., Lopes L.G., Menezes-Garcia Z., Ferreira A.V.M., Capettini L., Teixeira L.G., Lemos V.S. (2018). Sodium butyrate modulates adipocyte expansion, adipogenesis, and insulin receptor signaling by upregulation of ppar-gamma in obese apo e knockout mice. Nutrition.

[34] Matheus V.A., Monteiro L., Oliveira R.B., Maschio D.A., Collares-Buzato C.B. (2017). Butyrate reduces high-fat diet-induced metabolic alterations, hepatic steatosis and pancreatic beta cell and intestinal barrier dysfunctions in prediabetic mice. Exp. Biol. Med. (Maywood).

[35] Li H.P., Chen X., Li M.Q. (2013). Butyrate alleviates metabolic impairments and protects pancreatic beta cell function in pregnant mice with obesity. Int. J. Clin. Exp. Pathol..

[36] Kanuri G., Ladurner R., Skibovskaya J., Spruss A., Konigsrainer A., Bischoff S.C., Bergheim I. (2015). Expression of toll-like receptors 1–5 but not tlr 6–10 is elevated in livers of patients with non-alcoholic fatty liver disease. Liver Int..

[37] Mattace Raso G., Simeoli R., Russo R., Iacono A., Santoro A., Paciello O., Ferrante M.C., Canani R.B., Calignano A., Meli R. (2013). Effects of sodium butyrate and its synthetic amide derivative on liver inflammation and glucose tolerance in an animal model of steatosis induced by high fat diet. PLoS ONE.

[38] Bach Knudsen K.E., Laerke H.N., Hedemann M.S., Nielsen T.S., Ingerslev A.K., Gundelund Nielsen D.S., Theil P.K., Purup S., Hald S., Schioldan A.G. (2018). Impact of diet-modulated butyrate production on intestinal barrier function and inflammation. Nutrients.

[39] Correa-Oliveira R., Fachi J.L., Vieira A., Sato F.T., Vinolo M.A. (2016). Regulation of immune cell function by short-chain fatty acids. Clin. Transl. Immunol..

[40] Kawasaki T., Kawai T. (2014). Toll-like receptor signaling pathways. Front. Immunol..

[41] Wagnerberger S., Spruss A., Kanuri G., Volynets V., Stahl C., Bischoff S.C., Bergheim I. (2012). Toll-like receptors 1-9 are elevated in livers with fructose-induced hepatic steatosis. Br. J. Nutr..

[42] Weiss J., Barker J. (2018). Diverse pro-inflammatory endotoxin recognition systems of mammalian innate immunity. F1000Research.

[43] Iimuro Y., Frankenberg M.V., Arteel G.E., Bradford B.U., Wall C.A., Thurman R.G. (1997). Female rats exhibit greater susceptibility to early alcohol-induced liver injury than males. Am. J. Physiol..

[44] Deng M.H., Lopez G.-C., Lynch H.J., Wurtman R.J. (1991). Melatonin and its precursors in y79 human retinoblastoma cells: Effect of sodium butyrate. Brain Res..

[45] Wiechmann A.F., Burden M.A. (1999). Regulation of aa-nat and hiomt gene expression by butyrate and cyclic amp in y79 human retinoblastoma cells. J. Pineal Res..

[46] Haub S., Ritze Y., Ladel I., Saum K., Hubert A., Spruss A., Trautwein C., Bischoff S.C. (2011). Serotonin receptor type 3 antagonists improve obesity-associated fatty liver disease in mice. J. Pharmacol. Exp. Ther..

[47] Hatzis G., Ziakas P., Kavantzas N., Triantafyllou A., Sigalas P., Andreadou I., Ioannidis K., Chatzis S., Filis K., Papalampros A. (2013). Melatonin attenuates high fat diet-induced fatty liver disease in rats. World J. Hepatol..

[48] Celinski K., Konturek P.C., Slomka M., Cichoz-Lach H., Brzozowski T., Konturek S.J., Korolczuk A. (2014). Effects of treatment with melatonin and tryptophan on liver enzymes, parameters of fat metabolism and plasma levels of cytokines in patients with non-alcoholic fatty liver disease—14 months follow up. J. Physiol. Pharmacol..

[49] Chamanara M., Rashidian A., Mehr S.E., Dehpour A.R., Shirkohi R., Akbarian R., Abdollahi A., Rezayat S.M. (2019). Melatonin ameliorates tnbs-induced colitis in rats through the melatonin receptors: Involvement of tlr4/myd88/nf-kappab signalling pathway. Inflammopharmacology.

[50] Yao L., Lu P., Ling E.A. (2016). Melatonin suppresses toll like receptor 4-dependent caspase-3 signaling activation coupled with reduced production of proinflammatory mediators in hypoxic microglia. PLoS ONE.

[51] Hu Z.P., Fang X.L., Fang N., Wang X.B., Qian H.Y., Cao Z., Cheng Y., Wang B.N., Wang Y. (2013). Melatonin ameliorates vascular endothelial dysfunction, inflammation, and atherosclerosis by suppressing the tlr4/nf-kappab system in high-fat-fed rabbits. J. Pineal Res..

[52] Boffa L.C., Vidali G., Mann R.S., Allfrey V.G. (1978). Suppression of histone deacetylation in vivo and in vitro by sodium butyrate. J. Biol. Chem..

[53] Lumsden A.L., Martin A.M., Sun E.W., Schober G., Isaacs N.J., Pezos N., Wattchow D.A., de Fontgalland D., Rabbitt P., Hollington P. (2019). Sugar responses of human enterochromaffin cells depend on gut region, sex, and body mass. Nutrients.

[54] Haub S., Kanuri G., Volynets V., Brune T., Bischoff S.C., Bergheim I. (2010). Serotonin reuptake transporter (sert) plays a critical role in the onset of fructose-induced hepatic steatosis in mice. Am. J. Physiol. Gastrointest. Liver Physiol..

[55] Roshanravan N., Mahdavi R., Alizadeh E., Ghavami A., Rahbar Saadat Y., Mesri Alamdari N., Alipour S., Dastouri M.R., Ostadrahimi A. (2017). The effects of sodium butyrate and inulin supplementation on angiotensin signaling pathway via promotion of akkermansia muciniphila abundance in type 2 diabetes; a randomized, double-blind, placebo-controlled trial. J. Cardiovasc. Thorac. Res..

[56] Bouter K., Bakker G.J., Levin E., Hartstra A.V., Kootte R.S., Udayappan S.D., Katiraei S., Bahler L., Gilijamse P.W., Tremaroli V. (2018). Differential metabolic effects of oral butyrate treatment in lean versus metabolic syndrome subjects. Clin. Transl. Gastroenterol..

