# Supplementary material for: Oral Supplementation of Sodium Butyrate Attenuates the Progression of Non-Alcoholic Steatohepatitis
Source: Nutrients. 2020 Mar 30;12(4):951. doi: 10.3390/nu12040951 (PMC7231312; doi:10.3390/nu12040951)
Supplement: Supplementary file 1 [file nutrients-12-00951-s001.zip › Tables S_proofs.pdf]

Oral supplementation of sodium butyrate attenuates the progression of non-alcoholic steatohepatitis

Nutrients

Anja Baumann, Cheng Jun Jin, Annette Brandt, Cathrin Sellmann, Anika Nier, Markus Burkard, Sascha Venturelli, Ina Bergheim[#]

[#] **Corresponding author:** Ina Bergheim, Ph.D.
University of Vienna
Department of Nutritional Sciences
Molecular Nutritional Science
Althanstraße 14 (UZA II)
A-1090 Vienna
Email address: ina.bergheim@univie.ac.at

Table S1. Primer sequences used for real-time PCR.

	Forward (5´- 3´)	Reverse (5´- 3´)
18S	gtaacccgttgaaccccatt	ccatccaatcggtagtagcg
αSma	ctgacagaggcaccactgaa	catctccagagtccagcaca
Aanat	gcttctcctagtcccagcaccc	agcggaactcactggcaggg
Hiomt	ccctggcgtccaccttcctg	atggcggtgaaggggtcgtc
Il1β	tggctgtggagaagctgtgg	gtccgacagcacagaggcttt
Il6	ccacgccttccctacttca	tgcaagtgcatcatcgttgttc
iNos	cccctggaagtttctcttcaaagt	gattctggaacatctgtgctgtcc
Mtr1a	aatgccactcagcaggctccag	agcaggttgcccagaatgtcca
Mtr1b	agggctaccgtgcctgtcaa	aggtttgctgctaggcccact
Myd88	caaaagtggggtgcctttgc	aaatccacagtgcccccaga
Tgfβ	gtctgggaccctgcccctat	ttgcaggagcgcacaatcat
Tlr4	agccattgctgccaacatca	gctgcctcagcagggacttc
Tnfα	cagccaaccaggcagcgttcct	cctgccacaagcaggaatga

αSma, alpha smooth muscle actin; Aanat, serotonin N-acetyltransferase; Hiomt, hydroxyindole-O-methyltransferase; Il, interleukin; iNos, inducible nitric oxide synthase; Mtr, melatonin receptor; Myd88, myeloid differentiation primary response gene 88; Tgfβ, transforming growth factor beta; Tlr4, toll-like receptor 4; Tnfα, tumor necrosis factor alpha. Expressions were normalized to 18S mRNA expression.

Table S2. Effect of an oral supplementation of SoB on fibrosis markers in livers of mice with FFC-induced NASH.

	Diet groups				*p* (two-way ANOVA)		
	C	FFC	C + SoB	FFC + SoB	DExSoBE	SoBE	DE
**Sirius red staining (% of microscopic field)**	1.2 ± 0.2[a]	1.5 ± 0.4[a]	0.8 ± 0.1[a]	1.1 ± 0.1[a]	>0.05	>0.05	>0.05
***αSma* mRNA expression (% of control)**	100.0 ± 13.5[a]	177.5 ± 47.8[a]	192.1 ± 57.1[a]	262.3 ± 81.5[a]	>0.05	>0.05	>0.05
***Tgfβ* mRNA expression (% of control)**	100.0 ± 15.4[a]	129.8 ± 29.4[a]	82.0 ± 17.3[a]	157.2 ± 31.4[a]	>0.05	>0.05	<0.05

Data are shown as means ± SEM, n = 7-8. Data with different letters are significantly different, $p < 0.05$. αSma, alpha smooth muscle actin; C, control diet; DE, diet effect; DExSoBE, interaction between diet and SoB; FFC, fat-, fructose-, and cholesterol-rich diet; NASH, non-alcoholic steatohepatitis; SoB, sodium butyrate; SoBE; sodium butyrate effect; Tgfβ, transforming necrosis factor beta.

Table S3. Effect of SoB on NO_2^- concentration as well as mRNA expression of *iNos* and proinflammatory mediators in J774A.1 cells.

	Treatment groups				*p* (two-way ANOVA)		
	C	LPS	C + SoB	LPS + SoB	LPSExSoBE	SoBE	LPSE
**NO_2^- (% of control)**	100.0 ± 0[b]	6,379 ± 2,131[a]	76.6 ± 30.4[b]	4,240 ± 1,419[a]	>0.05	>0.05	<0.05
***iNos* mRNA expression (% of control)**	100.0 ± 0[b]	8,399 ± 2,284[a]	232.3 ± 37.5[c]	11,823 ± 1,790[a]	>0.05	<0.05	<0.05
***Tnfα* mRNA expression (% of control)**	100.0 ± 0[b]	607.8 ± 148.3[a]	137.9 ± 16.3[b]	698.4 ± 223.6[a]	>0.05	>0.05	<0.05
***Il1β* mRNA expression (% of control)**	100.0 ± 0[b]	116,264 ± 28,490[a]	151.0 ± 28.1[b]	315,263 ± 89,146[a]	>0.05	>0.05	<0.05
***Il6* mRNA expression (% of control)**	100.0 ± 0[b]	716 ± 131[a]	142.1 ± 20.3[b]	851.3 ± 210.8[a]	>0.05	>0.05	<0.05

Data are shown as means ± SEM, n = 5. Data with different letters are significantly different, $p < 0.05$. C, control; Il, interleukin; LPS, lipopolysaccharide; LPSE, LPS effect; LPSExSoBE, interaction between LPS and SoB; iNos, inducible nitric oxide synthase; NO, nitric oxide; SoB, sodium butyrate; SoBE; sodium butyrate effect; Tnfα, tumor necrosis factor alpha.

Table S4. Effect of SoB on mRNA expression of melatonin receptor, Aanat and Hiomt in small intestinal tissue of an everted gut sac model.

	Treatment groups		
	**C**	**3 mM SoB**	**6 mM SoB**
***Mtr1a* mRNA expression (% of control)**	100.0 ± 32.9[a]	134.7 ± 15.0[a]	178.0 ± 33.5[a]
***Mtr1b* mRNA expression (% of control)**	ND	ND	ND
***Aanat* mRNA expression (% of control)**	100.0 ± 15.3[a]	81.78 ± 11.0[a]	105 ± 9.7[a]
***Hiomt* mRNA expression (% of control)**	ND	ND	ND

Data are shown as means ± SEM, n = 5-6. Data with different letters are significantly different, $p < 0.05$. C, everted gut sacs incubated only in 1x Krebs-Henseleit-bicarbonate-buffer; Aanat, serotonin N-acetyltransferase; Hiomt, hydroxyindole-O-methyltransferase; *Mtr*, melatonin receptor; ND, not detectable; SoB, sodium butyrate.
