# Supplementary figures and images for: Oral Supplementation of Sodium Butyrate Attenuates the Progression of Non-Alcoholic Steatohepatitis
Source: Nutrients. 2020 Mar 30;12(4):951. doi: 10.3390/nu12040951 (PMC7231312; doi:10.3390/nu12040951)

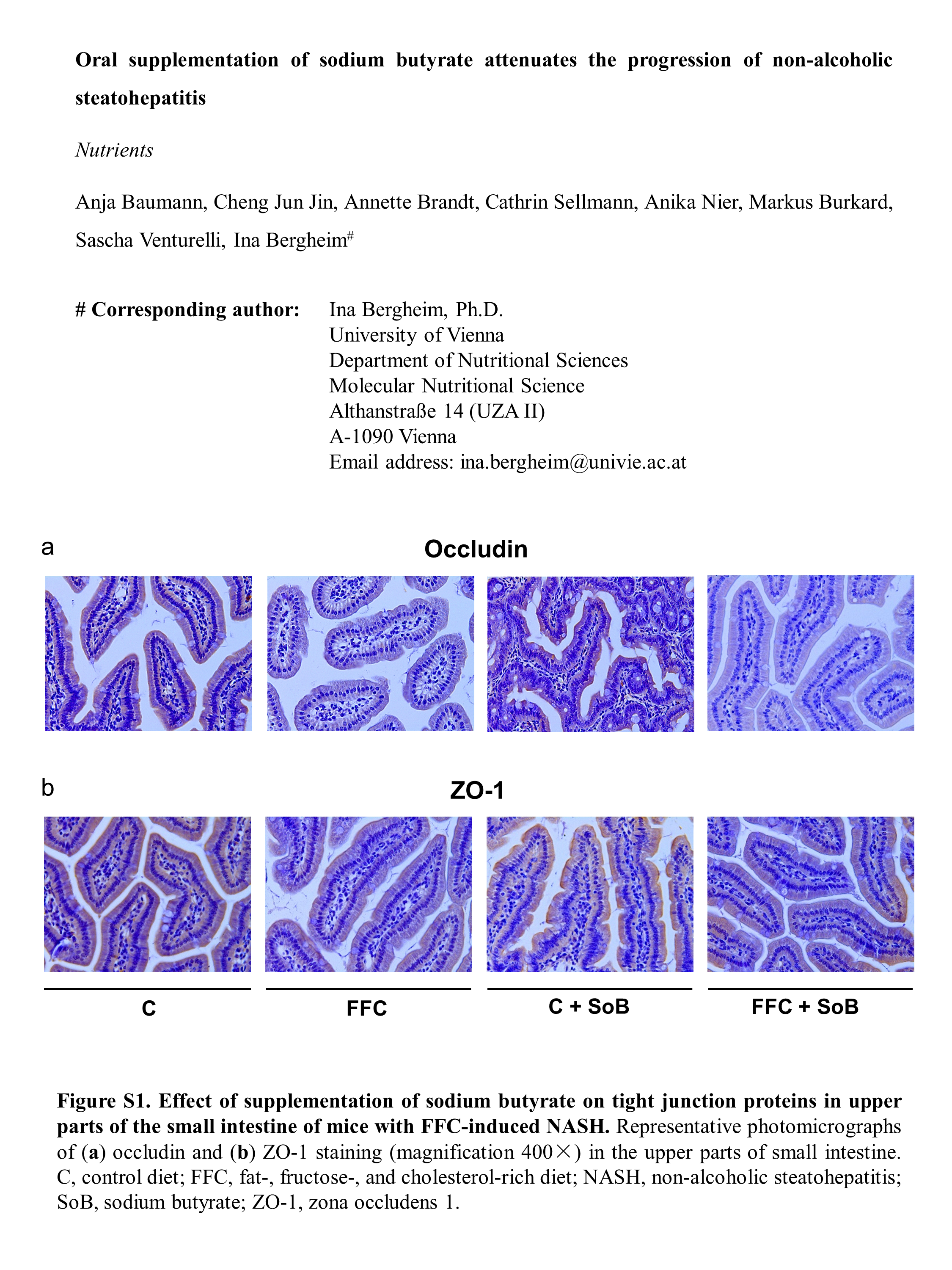

Supplement: Supplementary file 1 [file nutrients-12-00951-s001.zip › Figure S1_proofs.tif]
